# Muscle–tendon mechanics resolve the trade-off between energy-efficient and robust locomotion

**DOI:** 10.1098/rsbl.2025.0200

**Published:** 2025-11-05

**Authors:** Matthew Araz, Tobias Siebert, Alexander Badri-Spröwitz, Syn Schmitt, Daniel F. B. Haeufle

**Affiliations:** ^1^ Hertie Institute for Clinical Brain Research, University of Tübingen, Tübingen, Baden-Wuerttemberg, Germany; ^2^ Institute for Modelling and Simulation of Biomechanical Systems, University of Stuttgart, Stuttgart, Baden-Wuerttemberg, Germany; ^3^ Center for Bionic Intelligence Tuebingen Stuttgart (BITS), Tübingen-Stuttgart, Baden-Wuerttemberg, Germany; ^4^ Center of Integrative Neuroscience, University of Tübingen, Tübingen, Baden-Wuerttemberg, Germany; ^5^ Institute of Sport and Motion Science, University of Stuttgart, Stuttgart, Baden-Wuerttemberg, Germany; ^6^ Department of Mechanical Engineering, KU Leuven, Leuven, Flanders, Belgium

**Keywords:** muscle–tendon dynamics, mechanical work, energy efficiency, robustness, damping

## Abstract

Animals utilize elastic tendons in their limbs to store and release energy, reducing muscle effort and overall energy expenditure. At the same time, they navigate rough terrain dynamically without falling, despite significant neural delays. This ability allows them to achieve both robust and energy-efficient locomotion simultaneously—two properties often considered trade-offs in robotics. Through computational simulations, this study demonstrates how muscle–tendon mechanics can facilitate both energy-efficient and robust locomotion during perturbed vertical hopping across different muscle–tendon length configurations. Integrating muscle–tendon-like viscoelastic materials into legged robots may offer a solution to the previously perceived trade-off.

## 1. Introduction

Energy efficiency is an important aspect for animals to optimize during locomotion. Animals adopt energy-efficient strategies during steady legged locomotion [1,2]. To minimize metabolic energy expenditure, animals use compliant limbs and reduce the active muscle work via recycling energy through elastic tissues such as tendons and aponeuroses [3–6]. Energy storage and release is muscle-specific and depends primarily on tendon properties [7], the relative length of tendons in relation to the muscle–tendon unit (MTU) length [8], and the direction and timing of the flow of energy between the body, the elastic tendinous tissue and the contracting muscle [9]. Distal leg muscles that contribute to efficiency in bouncing gaits have comparatively longer and compliant tendons, compared with proximal muscles [6,10–12]. Due to the elastic properties of muscle–tendon complexes that power movement, locomotion mechanics are often explained with a spring-mass model [13–15]. Although spring-mass models effectively capture the fundamental mechanics of steady locomotion [14–17], they fail to explain robustness strategies animals employ to traverse natural terrains [18].

Animals can run robustly over rough terrain despite neural delays [19–21]. So called ‘preflexes’ are zero-delay force responses which can result from muscle intrinsic properties [22]. Preflexes can compensate for the neural delays, therefore help rejecting perturbations and contribute to robustness [23–25]. Muscle preflexes act within a few milliseconds, an order of magnitude faster than neural reflexes [26,27]. One mechanical origin for the preflex is the viscous-like damping behaviour of muscles, generated by the force–velocity relation, which may contribute to the dissipation of additional energy during perturbed locomotion [25,28,29]. Additionally, small but significant damping is also present in tendons, as shown in experiments [30] and proposed in simulation [31]. The importance of damping in locomotion stability has been indicated by studies that added damping to simple template models of compliant legs [18,32]. While damping benefits robustness, constantly active damping may cause unwanted energy dissipation, which forces the system to inject additional energy [33]. Thus, the robotics community often sees a trade-off between energy efficiency and robustness and prefers designs with minimal mechanical damping [33]. Several robotic studies influenced by muscle preflexes suggested perturbation-triggered damping strategies to resolve this trade-off [33]. However, how animals deal with this trade-off remains unclear.

In this study, we aimed to explore the trade-off between energy efficiency and robustness on the muscle level in locomotion. We compared two stimulation strategies, one that generates robust hopping and one that is optimized for energy-efficient steady locomotion. We hypothesized that an energy-efficient stimulation strategy would compromise robustness due to its energy-conserving nature, whereas a robust strategy would incur higher energy costs due to its reliance on dissipation. To our surprise, we found that the energy-efficient strategy is also more robust over perturbations. This indicates that muscle–tendon intrinsic mechanics might allow animals to be energy-efficient and robust simultaneously.

## 2. Methods

**Figure 1.**
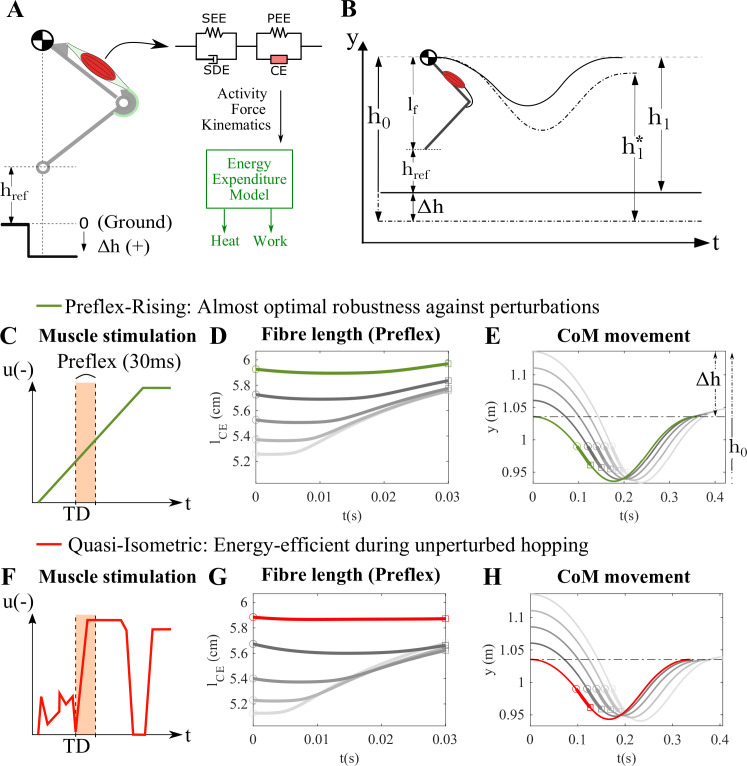
(A) Two-segment single-leg hopping model with a knee extensor muscle over uneven ground [28,34]. $h_{ref}$ presents the optimized reference hopping height that the preflex-rising strategy can generate. The four-element Hill-type muscle model proposed by Haeufle *et al.* [35] was implemented as the knee extensor muscle. The metabolic cost of hopping is calculated by the energy expenditure model proposed by Lichtwark & Wilson [3]. (B) Position y of the centre of mass (CoM) over time in unperturbed (periodic, solid line) and perturbed hopping simulations (dashed line). Each simulation included only a single vertical hopping cycle and was run separately for each perturbation case. $h_{0}$ is the release height, and $h_{1}$ is the return height after the hopping cycle. In unperturbed periodic hopping $h_{0}=h_{1}=h_{ref}+l_{f}$, where $h_{ref}$ is the reference hopping height, and $l_{f}$ is the fixed leg length during the flight phase. During perturbed hopping simulations (E and H), each perturbation (Δh) was introduced as $h_{0}=h_{\text{ref}}+l_{f}+\Delta h$, and the difference between the return height after perturbed hopping ($h_{1}^{*}$) and $h_{0}$ was used to quantify robustness. Panels (C–E) show the preflex-rising condition and panels (F–H) the quasi-isometric condition, illustrating muscle stimulation signals (C,F; green for preflex-rising and red for quasi-isometric), fibre length deviations during the preflex phase (D,G), and CoM trajectories during the hopping cycle for each perturbation case (E,H). Muscle stimulated with the preflex-rising strategy achieves almost optimal robustness against perturbations [28]. However, the muscle undergoes a stretch and shortening cycle during unperturbed hopping. On the contrary, the quasi-isometric strategy is optimized to minimize the muscle length change during the stance phase of unperturbed hopping to achieve the preflex-rising's hopping height in an energetically efficient way. A preflex time of 30 ms (orange bar) was assumed [26–28]. Green and red lines in fibre length and CoM movement plots show the unperturbed condition for the preflex-rising and quasi-isometric strategies, respectively. The grey scale gets lighter as the perturbation level increases. ◯ and □ represent the touchdown (TD) and end of preflex, respectively. Thick lines in the CoM movement plot show the preflex duration within the hopping cycle.

**Figure 2.**
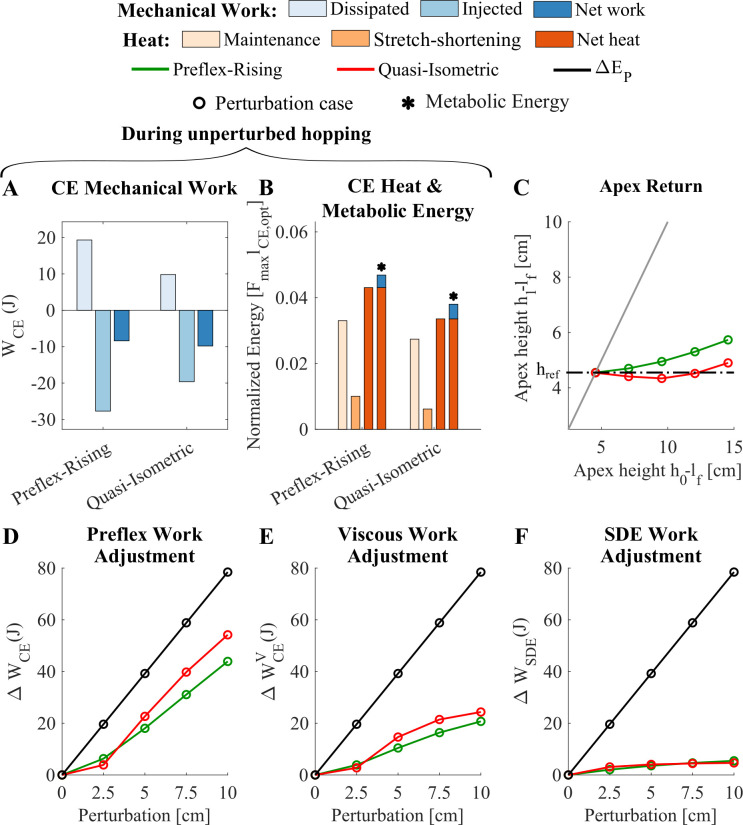
For γ=4 (A) contractile element work as the outcome of both preflex-rising and quasi-isometric stimulation strategies throughout the stance phase of unperturbed hopping. From lighter to darker blue bars show the dissipative, injected and net work, respectively. (B) The heat produced by CE during the stance phase of the unperturbed hopping, together with the heat components (maintenance and stretch-shortening heat) and the metabolic cost of the unperturbed hopping for both stimulation strategies. From light to dark orange bars show the maintenance, stretch-shortening and net heat produced by CE, respectively. The metabolic cost (*) is shown as the summation of net heat and work. (C) The apex return map indicates the robustness of the hopping model to step-down perturbations. For each release apex height $h_{0}-l_{f}$, the apex height after one hopping cycle $h_{1}-l_{f}$ is plotted (see also figure 1B). The grey solid diagonal line indicates periodic hopping: the reference hopping height ($h_{ref}$) at 4.55 cm leads to the same height at the end of the cycle. For higher release heights, $h_{1}$ is very close to the periodic height again, indicating that the perturbation is basically rejected within one hopping cycle, and this is true for both stimulation strategies. The dashed horizontal line indicates perfect rejection of any perturbation case within one cycle, representing the ideal robust solution. (D–F) Preflex work adjustment of CE, viscous work adjustment of CE, and SDE work adjustment in response to the perturbation, respectively. The black line represents the perturbation energy $\Delta E_{p}$, added to the body due to the step perturbation case (horizontal axis). '◯' represents the data for each perturbation case. If the work adjustment (coloured lines) is closer to the black line, the preflex adjusts better to the perturbation and dissipates more of the perturbation energy. (D) Preflex work of CE adjusts well to step-down perturbations, especially for the quasi-isometric strategy. (E) The viscous work of CE substantially contributes to the adaptation. (F) The adaptation of SDE work to the step-down perturbations is comparably small and saturates for larger perturbations.

Building on previous studies that investigate neuromechanical fundamentals of robust locomotion [28,34], we investigated the trade-off between energy efficiency and robustness with a muscle-actuated two-segment leg model. For detailed methodology, see electronic supplementary material, S.1. The model features only a single four-element Hill-type muscle model proposed by Haeufle *et al.* [35], implemented as a knee extensor, generating vertical hopping (figure 1A), to keep the analysis simple and maximize loading on the MTU [28]. We tested different muscle model parameters (for details see below), and the reference parameter set was based on previous models [28,34], see electronic supplementary material, S.2. Further, we implemented the energy expenditure model suggested by Lichtwark & Wilson [3] to calculate the metabolic cost of hopping (figure 1A, see electronic supplementary material, S.3 for model details and implementation). We investigated single hopping cycles, from initial apex (release height $h_{0}$) to the next apex ($h_{1}$) (figure 1B). Our reference behaviour was periodic hopping ($h_{0}=h_{1}=h_{ref}+l_{f}$, where $h_{ref}$ is the reference hopping height, and $l_{f}$ is the fixed leg length during the flight phase). Perturbations were introduced by increasing the release height $h_{0}=h_{\text{ref}}+l_{f}+\Delta h$ and evaluated again in single hopping cycles for each perturbed hopping height. The dynamics during the cycle and the apex at the end of the hopping cycle $h_{1}^{*}$ provided insights into the response to the perturbation. The combined picture of all perturbation cases in the ‘apex return map’ (figure 2C) shows the robustness of the model [29,34].

We analysed two feedforward stimulation strategies during unperturbed and perturbed vertical hopping: preflex-rising (figure 1C) and quasi-isometric (figure 1F). The preflex-rising strategy applies a linear ramp in muscle stimulation, previously shown to provide near-optimal robustness against step-down perturbations [28] and inspired by electromyography (EMG) measurements during *in vivo* perturbed hopping [36] and running [37]. However, this strategy does not prioritize energy efficiency during steady locomotion. To address this, we developed a quasi-isometric strategy optimized to minimize muscle length ($l_{CE}$) changes during the unperturbed condition (red line in figure 1G), thereby maximizing tendon energy storage and return for improved efficiency [38]. Both strategies were adjusted to achieve the same periodic hopping height under unperturbed conditions, ensuring equivalent impact conditions and enabling a fair comparison between robustness- and efficiency-oriented control against perturbations.

The model was tested against various step-down perturbations from 55% to 220% of $h_{ref}=4.55$cm (Δh=[2.5,5,7.5,10]cm) with both stimulation strategies. We used the decomposition algorithm proposed by Izzi *et al.* [28] to differentiate the velocity-, length- and activity-dependent force components of the contractile element during the preflex phase of each perturbed hopping. From this, we calculated total muscle work ($W_{CE}$) and its velocity-dependent contribution to capture viscous damping effects ($W_{CE}^{V}$). Preflex and viscous work adjustment ($\Delta W_{CE}$ and $\Delta W_{CE}^{V}$, respectively) were quantified as the change in muscle work between perturbed and unperturbed cases during the preflex phase (first 30 ms after touchdown (TD), orange areas in figure 1C,F) and compared against the additional perturbation energy ($\Delta E_{P}$). Full adjustment was defined as the condition in which the $\Delta W_{CE}$ exactly equalled the $\Delta E_{P}$, meaning the muscle fully dissipated the introduced perturbation energy. Furthermore, we evaluated the adjustment of the serial damping element (SDE) work ($\Delta W_{SDE}$) to assess the contribution of tendon dissipation to overall energy regulation.

To test the validity of our findings across different muscle–tendon configurations, we evaluated three fibre–tendon length (FTL) ratios (γ=[3.75,4,4.5]), as suggested by Mörl *et al*. [8]. The γ=4 condition served as the reference, and the other values were derived by modifying the reference condition. For each γ, we first optimized the periodic hopping height and stimulation start time of the preflex-rising strategy to match the activity level at touchdown ($a_{TD}=0.1$) with γ=4, ensuring comparable stimulation profiles across conditions. The quasi-isometric strategy was then optimized for each γ to achieve the same hopping height by minimizing muscle length changes during unperturbed hopping, enabling fair comparisons under perturbations. We restricted our analysis to these three values because, for higher ratios (γ>4.5), the relatively short muscle fibres could not generate sufficient force for stable periodic hopping, while for lower ratios (γ<3.75), the increased tendon stiffness amplified impact loads on the muscle and prevented distinct quasi-isometric solutions.

## 3. Results

Across different FTL ratios, our optimization strategy (quasi-isometric stimulation) reduced the muscle length change during the stance phase compared with the preflex-rising strategy. As expected, this resulted in more energy-efficient steady-state locomotion at all FTL ratios. However, we observed the highest metabolic energy difference between preflex-rising and quasi-isometric strategies at the reference FTL condition γ=4. As we deviated from the reference value, the energetic difference is reduced. Thus, the results focused on the reference condition γ=4 (see electronic supplementary material, figure S7 for the results of γ=3.75 and electronic supplementary material, figure S8 for the γ=4.5). We observed a 50% reduction of dissipated mechanical work (figure 2A) and, consequently, the muscle needed to inject 30% less mechanical work and 19% less metabolic energy (‘*’ in figure 2B). The quasi-isometric strategy lowered the metabolic cost by reducing the maintenance heat ( $Q_{m}$) by 17% and stretch-shortening heat ( $Q_{sl}$ ) by 38%, resulting in a 22% reduction in net heat (figure 2B). This is a substantially more energy-efficient strategy—exactly what we aimed for.

Surprisingly, this energy-efficient strategy does not come with a loss in robustness. Instead, the quasi-isometric strategy provides slightly better robustness. This can be observed in the apex return map (figure 2C), where the quasi-isometric strategy (red line) had a better alignment with the dashed black line, which represents ideal robustness, compared with the preflex-rising strategy (green line). It can also be seen in figure 1E,H (also electronic supplementary material, figure S1), which shows that the hopping height at the end of the perturbed hopping cycle is almost perfectly aligned again with the reference hopping height $h_{\text{ref}}$. The main reason behind the improved robustness is that the quasi-isometric strategy (red lines) better aligned the preflex work adjustment ($\Delta W_{CE}$, figure 2D) and muscle viscous adjustment ($\Delta W_{CE}^{V}$, figure 2E) to the perturbation energy ($\Delta E_{P}$, black line in figure 2D–F) than the preflex-rising strategy (green lines). A decomposition of the CE force reveals that a significant portion of the muscle’s preflex adjustment arises from viscous forces generated by the muscle’s force–velocity relationship (figure 2E). Meanwhile, the adjustment of SDE ($\Delta W_{SDE}$) was observed to be quite small and not diverse between stimulation strategies (figure 2F). Our findings indicate that muscle–tendon mechanics may enable achieving both energy-efficient and robust locomotion without relying solely on task-level stability.

## 4. Discussion

In this study, we showed that muscle–tendon intrinsic properties are crucial for achieving energy-efficient and robust locomotion simultaneously. We optimized a single-leg hopper’s muscle stimulation pattern to minimize muscle length change during unperturbed hopping. We then tested the model’s response to step-down perturbations. Our results for the reference γ condition demonstrated that the 19% more energy-efficient hopping with the quasi-isometric muscle stimulation strategy does not come at the cost of reduced robustness. Instead, it improved the robustness slightly beyond the preflex-rising condition, which was already near the optimal robustness (figure 2C). Similar outcomes also observed for the other γ conditions. However, as we deviated from the reference γ condition, the energetic benefit of the quasi-isometric strategy over the preflex-rising strategy reduced; showing that there is a certain FTL ratio at which muscle can perform with the highest efficiency [3]. Thus, the discussion is built based on the results of the reference γ condition.

Previous studies have shown that legged animals use energetically efficient strategies, cycling energy through tendons to reduce muscle work and lower metabolic cost during unperturbed locomotion [3–6]. Consistent with these findings, our results indicate that minimizing muscle length change leads to more energy-efficient hopping (‘*’ in figure 2B). The quasi-isometric stimulation strategy reduces the dissipative work throughout the stance phase (figure 2A). Thus, less energy is needed to be injected by the contractile element to achieve the periodic hopping height (figure 2A). Together with the lower mechanical work, the quasi-isometric stimulation strategy also lowers heat production (figure 2B). As expected, less change in muscle length reduced the stretch-shortening heat ($Q_{sl}$) generation (figure 2B). Also, the maintenance heat ($Q_{m}$) is slightly lower with the quasi-isometric strategy (figure 2B) even though activity levels throughout the stance phase are higher than in the preflex-rising condition. Our results indicate that lower maintenance heat arises from the shorter duration of active force generation. In the quasi-isometric strategy, a sharp rise in activation at the early stance enabled the contractile element to stiffen quickly and load the tendon together with the external load, which causes more rapid release of stored energy, resulting in a shorter stance duration (electronic supplementary material, figure S1). Despite higher activation levels, this reduced the time of active force production and lowered maintenance heat per hop. These findings align with previous work highlighting the importance of active force duration in metabolic cost [39].

Our findings align with *in vivo* human hopping studies showing that increasing hopping frequency enhances tendon contribution to positive work and lowers metabolic cost [40,41]. These studies identified resonance frequency as the most energy-efficient condition, with minimal muscle and maximal tendon work, while deviations increased muscle work and metabolic cost [41]. Although the quasi-isometric strategy reduced muscle work and heat solely by minimizing muscle length change, incorporating resonance-based optimization could further enhance efficiency.

Surprisingly, the quasi-isometric strategy not only enhanced energy efficiency but also improved robustness in response to step-down perturbations. Our results demonstrate that the quasi-isometric strategy enabled better recovery from perturbations (figure 2C) due to better preflex adjustment (figure 2D, which can also be seen in work-loops electronic supplementary material, figure S2, and power-time series of CE electronic supplementary material, figure S6A,B) together with slightly improved viscous adjustment (figure 2E and can be seen in power-time series of viscous CE force electronic supplementary material, figure S6C,D). But why did the quasi-isometric strategy not perform worse in terms of robustness compared with the preflex-rising strategy, which was already near the optimal robustness? During unsteady locomotion, step-down perturbations introduce a delay in contact, which leads to a higher activity level at touchdown due to the sharp rising profile of the quasi-isometric strategy. This results in greater muscle force (electronic supplementary material, figure S3A,B), increased concentric contraction during the flight phase (electronic supplementary material, figure S4A,B), and a stiffer tendon at impact (electronic supplementary material, figures S3E,F and S4C,D) due to the larger stretch of the nonlinear force–length characteristic of the serial elastic element (SEE). As a result, the tendon rapidly transmits impact forces to the highly activated muscle, causing a faster switch from concentric to eccentric contraction resulting in larger velocities (electronic supplementary material, figure S5), dissipating the excessive energy from the step-down perturbation (electronic supplementary material, figures S2 and S6A–D). Although the preflex-rising strategy also has a rising stimulation profile, the ramp increase is not as sharp as the quasi-isometric strategy. Thus, the increase in muscle activity is insufficient to stiffen the muscle enough to resist the external loading, causing energy dissipation by CE during the preflex phase of unperturbed hopping (can be seen in work-loops electronic supplementary material, figure S2A and power-time traces electronic supplementary material, figure S6A). Besides, a slower increase of stimulation causes lower activity levels compared with the quasi-isometric strategy during the perturbed hopping, resulting in lower energy dissipation (can be seen in work-loops electronic supplementary material, figure S2 and power-time traces electronic supplementary material, figure S6A–D). Thus, the preflex adjustment by the preflex-rising strategy was less effective than the quasi-isometric strategy. This shows that minimizing muscle work by reducing muscle length changes allowed for better preflex adjustments (figure 2D,E), which led to both energy-efficient and robust locomotion simultaneously. Additionally, the quasi-isometric strategy also enabled more effective use of energy dissipation from CE and MTU through the stance phase over the step-down perturbations (electronic supplementary material, figures S2 and S6) with lower metabolic cost. This shows that minimizing damping during unperturbed hopping yields better adjustment of the dissipative work to the perturbation energy (figure 2D,E).

Energy efficiency and robustness are two critical aspects of legged locomotion for animals’ survival. *In vivo* studies have shown that animals can reject perturbations relying only on feed-forward signals optimized for efficiency [19], yet early compliant-limb models suggested a trade-off between robust and energy-efficient locomotion [18,32,33]. Studies on unsteady locomotion suggested that muscle intrinsic mechanics provide rapid mechanical feedback for perturbation rejection despite neural delays [20,23–25]. Muscle-actuated compliant limb models indicated that the necessity of muscle force–velocity relation for the dynamic stability [29], but by neglecting tendon mechanics, these models fail to explain how robustness and efficiency are achieved simultaneously. We found that both muscle and tendon mechanics are essential for achieving energy-efficient and robust locomotion simultaneously. By decoupling MTU and CE lengthening, tendon elasticity reduced muscle work during unperturbed hopping while improving preflex work adjustment and viscous dissipation against perturbations [42]. As a result, the hopper leg recovered more effectively from step-down perturbations with the energy-efficient strategy. Thus, energy-efficient use of tendon mechanics not only lowers metabolic cost but also enhances muscular damping, allowing robust locomotion with solely feed-forward control optimized for energy efficiency.

Like animals, achieving energy-efficient and robust locomotion is also crucial for legged robots. In legged robotics, this goal has inspired the use of passive mechanical systems based on animal morphology. Recent studies have proposed perturbation-triggered damping—where damping is activated only when a perturbation occurs—to improve efficiency and robustness compared with constant damping [32,33]. However, this mechanism only mimics the dissipative characteristics of muscles and neglects the tendon’s energetic benefits. Our findings extend this concept by showing that intrinsic muscle–tendon mechanics naturally provide a similar function: recycling energy via the tendon minimizes muscular dissipation during steady hopping, while muscle dissipation is more effectively adjusted during perturbations. This suggests that incorporating viscoelastic materials replicating muscle–tendon interactions, rather than mimicking muscle or tendon mechanics alone, could offer a more effective solution for energy-efficient and robust robot locomotion.

In this study, we demonstrated the importance of muscle–tendon intrinsic mechanics in achieving energy-efficient and robust locomotion. Across different FTL ratios, the hopper leg was more robust when stimulation minimized muscle length changes, as this not only enabled tendon-based energy cycling to reduce metabolic cost but also enhanced tunable muscular damping for perturbation rejection. Yet, our study has limitations that need to be taken into consideration. In this study, we used a simplified leg model with a single MTU, modelled with a phenomenological Hill-type muscle model, to focus on the muscle’s intrinsic adjustment capacity. Although Hill-type muscle models have known limitations in predicting muscle force [26,43,44], particularly during eccentric contractions [26,45], Araz *et al.* [26] showed that the model of Haeufle *et al.* [35] predicted preflex work of skinned fibres under realistic hopping trajectories within an acceptable accuracy. Therefore, despite its limitations, we believe that the Hill-type muscle model can provide meaningful insight into the role of intrinsic muscle–tendon mechanics on the trade-off between energy efficiency and robustness in biological locomotion. Furthermore, although Lichtwark & Wilson [3]’s energy expenditure model has been shown to overestimate the heat values [46], we choose this model since it also includes the heat production during eccentric contractions. As we were interested in the relative change of heat between preflex-rising and quasi-isometric strategies, instead of the actual values, the model choice seems appropriate to us.

## Data Availability

All Matlab Simulink files needed to replicate the manuscript results and figures are available in the FDAT Research Data Repository of the University of Tübingen https://doi.org/10.57754/FDAT.1yfjh-2p259 [47]. Supplementary material is available online [48].
